# Ambient-Pressure Near-Edge X‑ray Absorption Fine Structure Study of the Photothermal Water Splitting Process on the Cu:CeO_2_ Nanostructure Surface

**DOI:** 10.1021/acsanm.6c00868

**Published:** 2026-05-13

**Authors:** Eleonora Spurio, Silvia Mauri, Samuele Pelatti, Mario Leopoldo Rivera-Salazar, Sergio D’Addato, Piero Torelli, Paola Luches, Stefania Benedetti

**Affiliations:** † Istituto Nanoscienze – CNR, S3, Modena 41125, Italy; ‡ CNR-IOM, Laboratorio TASC, Basovizza, Trieste 34149, Italy; § MAX IV Laboratory, 5193 Lund University, SE-221 00 Lund, Sweden; ∥ Dipartimento di Fisica, 9315 Università di Trieste, Trieste 34127, Italy; ⊥ Dipartimento di Scienze Fisiche, Informatiche, Matematiche, 9306 Università di Modena e Reggio Emilia, Modena 41125, Italy

**Keywords:** Cu-doped CeO_2_, photothermal water splitting, ambient-pressure NEXAFS, hydrogen production, gas chromatography, cerium oxide

## Abstract

The modification of the electronic properties of CeO_2_ thin films through Cu doping is a promising approach to enhancing their performance in photoinduced water splitting (WS). In this study, undoped and Cu-doped CeO_2_ films of 5 nm thickness with 5 and 11% Cu atomic concentrations were characterized under ambient pressure conditions by near-edge X-ray absorption fine structure (NEXAFS) to provide insights into the film modification and the water splitting process during exposure to water and to laser light at different temperatures. The analysis of NEXAFS data, acquired at the Ce M_5_ and Cu L_3_ edges, reveals temperature-dependent changes in the oxidation states of Ce and Cu. Notably, a temperature-dependent evolution of the Ce^3+^ concentration is observed during water exposure, accompanied by spectral changes consistent with a partial reduction of Cu^2+^ to Cu^1+^ in the film with the highest dopant concentration. The ambient-pressure NEXAFS measurements highlight the role of Cu dopant ions in modifying the electronic structure of CeO_2_ and the catalytic response in the presence of water and illumination, enabling more efficient hydrogen production. In parallel, micro gas chromatography analysis revealed a marked increase in hydrogen production from the 11% Cu-doped CeO_2_ film compared to the undoped CeO_2_ and the 5% Cu-doped films. These results provide crucial insights into the structure–function relationship in Cu-doped CeO_2_, offering pathways to optimize the design of materials for hydrogen production and related applications.

## Graphical abstract

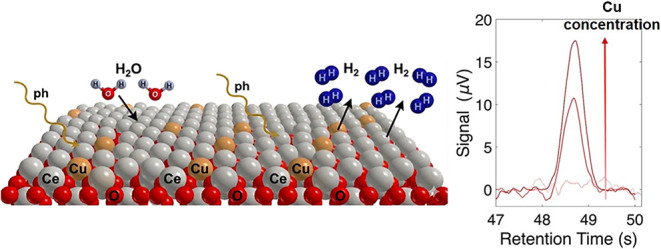

## 1 Introduction

Photocatalytic water splitting (WS) is a promising way to produce green hydrogen, exploiting only light irradiation and a catalyst to dissociate water. Catalyst materials based on reducible oxides are particularly intriguing due to their capability to store and release oxygen in response to environmental modifications.^1^ Among these materials, cerium oxide has drawn attention thanks to the stability of its cation in two oxidation states, Ce^4+^ and Ce^3+^, and the ease of forming oxygen vacancies, which enhances its catalytic activity toward various surface chemical reactions.^2,3^ Moreover, cerium oxide is resistant to chemical and photocorrosion, and it is characterized by strong light absorption in the UV region.^4^ The O 2p → Ce 4f optical band gap of stoichiometric CeO_2_ is typically reported as 3.0–3.5 eV,^5–7^ although some of us recently proposed a 4 eV optical gap for stoichiometric samples, based on steady-state and ultrafast transient absorption spectroscopy results.^8^ However, the onset of absorption is typically shifted toward lower energies by increasing the concentration of oxygen vacancies and/or by doping. It has been observed that Ce substitution with metallic ions can efficiently increase the catalytic activity of cerium oxide.^3,9–11^ The metal inclusion has also been demonstrated to enhance the photochemical activity by narrowing the band gap, suppressing the charge carrier recombination, facilitating the exchange of oxygen and electrons with the surroundings, reducing the reaction energy barriers, and promoting the formation of oxygen vacancies.^12–15^ Dopant atoms generally induce local distortions in the crystal lattice, which are typically associated with a reduction in the oxygen vacancy formation energy in their vicinity. Additionally, when the dopant valence differs from the Ce^4+^ state in CeO_2_, this reduction is further influenced by modifications to the electronic structure of the material.^16^ Among the different transition metals used as dopants, Cu has been shown to be a valid alternative to noble metals, thanks to its unique catalytic features and lower cost.^12,13,17–21^ The inclusion of aliovalent dopant ions, such as Cu, in ceria films has been demonstrated to facilitate the reduction processes,^22^ the formation of oxygen vacancies,^3,11,12,23^ and the stabilization of active intermediates at the interface.^24^ However, despite significant progress, the precise molecular mechanisms behind this synergism and the role of Cu in its different oxidation states and of oxygen vacancies remain unclear due to the complexity of the copper–ceria interaction and the challenges associated with the application of analytical techniques in operando conditions. A detailed understanding of the structural and electronic modifications induced by Cu at the atomic level is crucial to clarify how reactions evolve on the cerium oxide surface. Previous studies demonstrated that when Cu is introduced as a dopant in CeO_2_ films, it is generally in the Cu^2+^ state^25^ but can undergo reduction cycles that involve an intermediate Cu^1+^ state,^26^ the latter being the most catalytically active species. It was demonstrated that the introduction of Cu dopants in CeO_2_ thin films promotes H_2_ dissociation and CeO_2_ reduction, allowing ceria to become reactive at lower temperatures.^11^ The study of the electronic modifications by near-edge X-ray absorption fine structure (NEXAFS) in ambient pressure during the exposure to gas has proved to give interesting information on the chemical processes in realistic conditions.^27^ A recent study by some of the authors^3^ exploited a combination of ambient-pressure NEXAFS (AP-NEXAFS) investigation and micro gas chromatography to correlate the changes of the chemical state of Cu and Ce cations in Cu:CeO_2_ films with water desorption in real time during temperature treatments in an ambient pressure of hydrogen. In this work, we present a detailed study of the photothermal water splitting process on the Cu:CeO_2_ surface (compared to pure oxide) and the electronic modifications occurring in the nanometric film in ambient pressure conditions, to clarify the role of the dopant and oxygen vacancies in the water dissociation and H_2_ formation. In contrast to our previous work performed under a reducing H_2_ atmosphere,^3^ the present study is carried out in the presence of water under ambient pressure and UV illumination. This difference in the reaction environment leads to a substantially different behavior: while in H_2_, a progressive reduction of the oxide is observed, in H_2_O, the system evolves under competing reduction and oxidation processes associated with water dissociation and hydrogen formation, with water promoting the formation of oxygen vacancies at low temperatures. As a result, the electronic structure evolution differs significantly from that observed under purely reducing conditions. This study is crucial for the optimization of the material and to improve catalyst efficiency in the production of green hydrogen.

## 2 Experimental Section

The samples investigated in this work are pure and Cu-doped cerium oxide films of 5 nm nominal thickness, deposited on MgO(001) 2-side polished substrates at room temperature (RT). For the doped samples, 5 and 11 atom % Cu-doped CeO_2_, the doping concentration is defined as the atomic ratio Cu/(Cu + Ce). Before the deposition, the MgO substrates were cleaned by a 5 min bath in boiling acetone and by two subsequent ultrasonic baths in boiling acetone and isopropanol for 3 min each, followed by ultrahigh-vacuum (UHV) annealing to 770 K and treated by short sputtering with Ar^+^ ions to remove surface contaminants that prevent film adhesion. The samples were grown and characterized in the apparatus described in ref 28, composed of two connected UHV chambers. The first one is used for substrate preparation and characterization through X-ray photoemission spectroscopy (XPS), while the second one is equipped with evaporators, gas lines, and a quartz microbalance and it is used for sample deposition. The films characterized here were grown following the procedure described in refs 3,11 . The undoped ceria films were deposited by reactive molecular beam epitaxy, evaporating Ce from a high-temperature effusion cell, while the doped samples were grown by codeposition of Ce and Cu atoms. O_2_ was introduced during the deposition by a nozzle with a partial pressure of 1 × 10^–7^ mbar. The dopant concentration was determined by the evaporation rate of Ce and Cu, measured by the quartz microbalance in the deposition chamber, and by *in situ* XPS measured after the growth. To ensure complete oxidation of Ce, the films were annealed to 770 K in 1 × 10^–7^ mbar of O_2_ after deposition, and the final composition of the film was checked by XPS. After the sample deposition and XPS characterization, the optical absorptance of the samples was investigated using UV–vis optical spectrophotometry. The setup includes a xenon lamp providing white, nonpolarized light, an ORIEL-MS257 monochromator, a polarizer, and a silicon photodetector. The absorptance (*A*) is calculated as *A* = 1 – (*T* + *R*), where *T* and *R* represent the transmittance and reflectance, respectively (i.e., the fractions of light transmitted and reflected). These measurements were conducted with the incident photon beam at an angle of 22° relative to the sample surface normal.

The NEXAFS measurements were performed at the APE-HE beamline of the Elettra synchrotron radiation source in the reaction cell described in refs 3,27 . Before starting the experiment, the samples were oxidized by heating to 573 K in a mixture of O_2_/He (10/40 sccm, 1 bar total pressure) to remove the surface contaminants and restore surface oxidation.

The modifications in the electronic structure were studied by measuring the NEXAFS spectra at the Ce M_4,5_ and Cu L_2,3_ edges in total electron yield, during the exposure of the samples to 1 bar of water flux, about 10 sccm provided by a bubbler in He carrier, in the reaction cell, both in the dark and with laser illumination at temperatures between 373 and 573 K. The NEXAFS spectra were acquired in steps of 50 K. A laser wavelength of 375 nm was chosen to be at the onset of the absorption peak related to the optical gap in the samples determined by UV–vis spectra shown in Figure 2. In order to follow the sample evolution caused by the interaction with the laser and with water in the reaction cell, the Ce M_5_ and Cu L_3_ NEXAFS spectra were acquired after each step of the following sequence (see schematics in Figure 1): 1. The sample at low temperature (373 K) in a He atmosphere, with no H_2_O and no laser illumination (water off, laser off); 2. Turning on the laser without introducing water and keeping the sample at 373 K (water off, laser on); 3. Turning off the laser and introducing water, keeping the sample at 373 K (water on, laser off); 4. Turning on the laser and fluxing water, keeping the sample at 373 K (water on, laser on); 5. Starting the temperature ramp and always fluxing water: 5.1. without the laser (water on, laser off) and 5.2. with the laser (water on, laser on).

**Figure 1:**
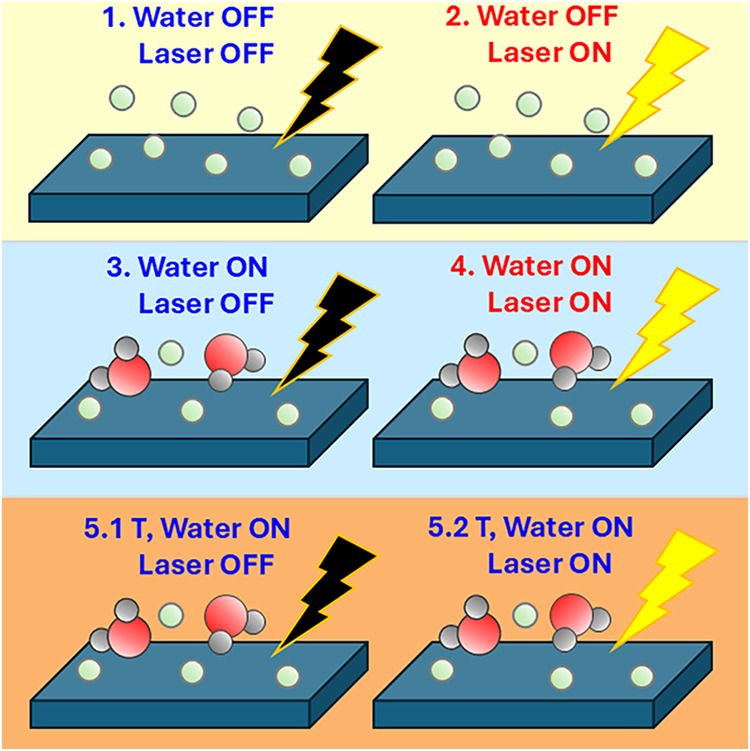
Schematics of the procedure followed during the experiment.

Micro gas chromatography (micro-GC) data were collected on identical samples under the same conditions in a N_2_ carrier to identify H_2_ production in the reaction cell as a function of temperature and avoid He and H_2_ signal superposition. The products of the water splitting reaction were detected by an Agilent 990 micro-GC equipped with two chromatographic columns connected through the output of the gas line to the reaction cell. A Poraplot chromatographic column, heated to 380 K, using N_2_ as the carrier gas, has been used, acquiring the chromatograms continuously during the reaction in a time window of 180 s. The uncertainty associated with the micro-GC results was calculated as the maximum deviation from zero observed where no signal was expected. This approach was used to conservatively estimate the instrumental noise level and quantify the uncertainty of the absorbance values. The micro-GC measurements were not calibrated for the absolute quantification of H_2_ concentration. Therefore, the reported signals are used to compare the relative trends in hydrogen production among the different samples and conditions.

The Ce M_5_ NEXAFS spectra acquired at the different temperatures and with/without the laser illumination were analyzed with a procedure similar to the one followed in ref 3. The fitting of the Ce M_5_-edge spectra was conducted after aligning the edge to a reference powder measured concurrently with the film, to eliminate small drifts of the synchrotron photon energy, and after subtracting a linear pre-edge background. The reference Ce M_5_ spectra on CeO_2_ and Ce_2_O_3_ were obtained on commercial powders from Umicore. A linear combination of the two reference spectra for the two oxidation states normalized to the M_5_ area was used to fit the NEXAFS spectra. The fitting coefficients, *A*_Ce^3+^_ and *B*_Ce^4+^_, allowed us to estimate the Ce^3+^ concentration under the different conditions as $\frac{A_{Ce^{3+}}}{B_{Ce^{4+}}+A_{Ce^{3+}}}$ . The uncertainties were assumed as one standard deviation and were all estimated to be 1% of the extracted Ce^3+^ concentration.

The intensity of the Cu L_3_-edge NEXAFS signal was too low to be properly fitted using reference signals because of the low concentration of Cu in the samples combined with the low photoionization cross-section of the Cu L_3_-edge, especially in the case of Cu^1+^ and Cu^0^.^3,29,30^ However, the variations of the signal at different temperatures and under laser illumination were qualitatively analyzed to retrieve information on the oxidation state of the Cu dopant.

## 3 Results

Figure 2 shows the optical absorptance spectra of the undoped (purple), Cu:CeO_2_ 5 atom % (green), and Cu:CeO_2_ 11 atom % (yellow), together with the absorptance of the bare MgO substrate for comparison, in black. The optical absorptance of the three films is almost identical, with the main absorptance peak at ∼320 nm and the absorptance edge ascribed to the band gap excitation of cerium oxide^8,31^ at about 400 nm. Besides the main absorptance peak, another two features can be observed in the spectrum: a broad and weak absorptance band in the visible region, which is visible for all the four spectra and that is ascribed to a mild defect-assisted absorptance of the substrate (black curve in Figure 2), and a shoulder between 350 and 400 nm, which is ascribed to the presence of a low concentration of oxygen vacancies in the film.^6,32^ The optical band gap of the three films (undoped, low and high dopant concentrations) was determined as described in ref 33, i.e., by calculating the second derivative of the absorptance as a function of wavelength. This method is particularly efficient when two or more light-absorbing substances are present: the positive peak provides the position of band gap energies for the film. The plot of d^2^ *A*/dλ^2^ is shown in the inset in Figure 2: the position of the positive peak is for all samples at ∼338 nm (3.67 eV).

**Figure 2:**
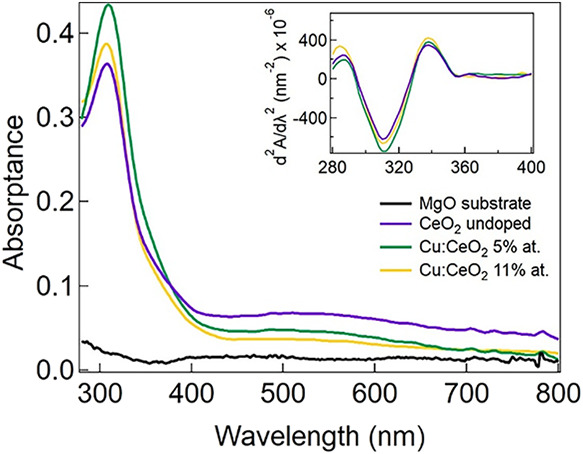
UV–vis absorptance. Inset: the second derivative of the absorptance with respect to λ, d^2^ *A*/dλ^2^.

These observations suggest that Cu is well-dispersed within the CeO_2_ lattice without forming large CuO or Cu_2_O clusters or secondary phases, as such phases would introduce additional absorption features and modify the second derivative of the absorptance. Based on these results, a laser wavelength of 375 nm has been chosen for the experiment. The Ce 3d XPS spectra shown in Figure S1 allow us to evaluate the chemical state of the Ce ions and thus the oxidation state of Ce cations within the films. As better explained in the SI, the Ce 3d XPS spectra have been fitted to extract the film stoichiometry, showing that all films show a dominant CeO_2_ stoichiometry, with a Ce^3+^ concentration below the detection limit, supporting the fact that doping does not alter the Ce oxidation state. Figure 3a shows the Ce M_5_ edge NEXAFS spectra for the undoped CeO_2_ film acquired at different temperatures with the laser on and off. After the oxidation, prior to the water and laser exposure, at 373 K (H_2_O off, laser off), the spectrum shows a dominant peak at ∼881.5 eV and a satellite at ∼887 eV. These features correspond to the ones in the Ce^4+^ reference spectrum (Figure S2a),^3^ related to the 3d → 4f state transition with multiplet splitting effects.^34^ When the sample is illuminated (H_2_O off, laser on), the ceria film undergoes a slight reduction with respect to the initial conditions, as highlighted by the appearance of the two features on the low-photon energy side of the edge and ascribed to the formation of Ce^3+^ ions.^3^ When the laser is turned off and water is fluxed, the concentration of the Ce^3+^ ions remains unchanged. Increasing the temperature leads to a faint increase in the peak at 879 eV up to 473 K that corresponds to an increase in the fraction of reduced ions. The concentration of Ce^3+^ ions at different temperatures and with/without the laser illumination for the undoped film, obtained by fitting with a linear combination of spectra from reference samples, is reported in Figure 4a. For each sample, the graph has been divided into five panels. The first panel reports the Ce^3+^ concentration (with and without the laser) before fluxing water in the reaction cell; the other panels report the Ce^3+^ concentration in the water flux at increasing temperature in the dark (blue dots) and under light (red dots). Ce^3+^ fractions carry ±1% uncertainty (from fit standard deviations), below the marker size in Figure 4, accounting for noise and fitting procedures. This uncertainty indicates the precision of the results obtained from the fitting procedure. On the contrary, the accuracy is lower, due to the approximations that are assumed (e.g., on the background removal, given its unknown shape, or the possible presence of satellites). However, here, we are interested in the relative values and in the comparison between different samples and not in the absolute values. The undoped film is almost completely oxidized at the beginning of the ramp (Figure 4a), and it undergoes a first, mild reduction when the sample is first illuminated. The fraction of Ce^3+^ ions remains unchanged when water is introduced in the reaction chamber, independently of the sample illumination. The concentration of the reduced ions increases to 17% when the temperature reaches 473 K, independently of the presence of light, and it slightly decreases to 14% at 523 K, remaining the same also at higher temperature. It can be concluded that the interaction of the fully oxidized film with the laser causes the reduction of part of the Ce ions at low temperature, while the effect of the laser is negligible in the temperature ramp in water, where the main effects can be ascribed to the temperature increase.

**Figure 3:**
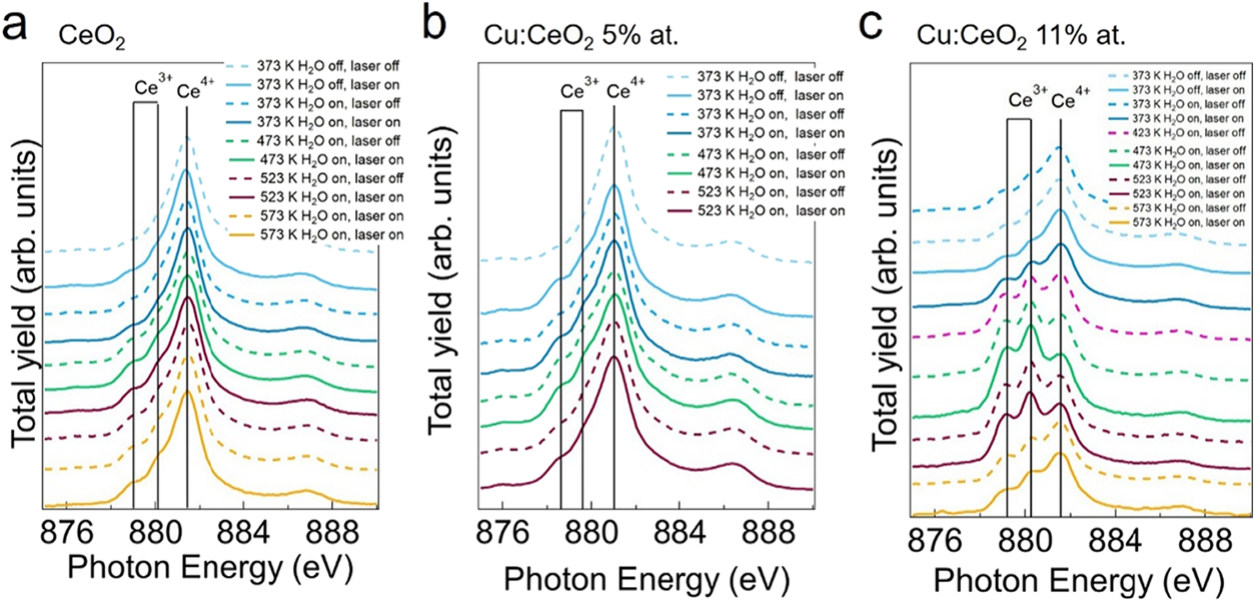
Ce M_5_ edge NEXAFS spectra for (a) undoped CeO_2_, (b) Cu:CeO_2_ 5 %, and (c) 11 % films acquired at different temperatures in H_2_O flux. The measurements performed with the laser on are reported in the figure as solid lines, while the ones in the dark are reported as dashed lines.

**Figure 4:**
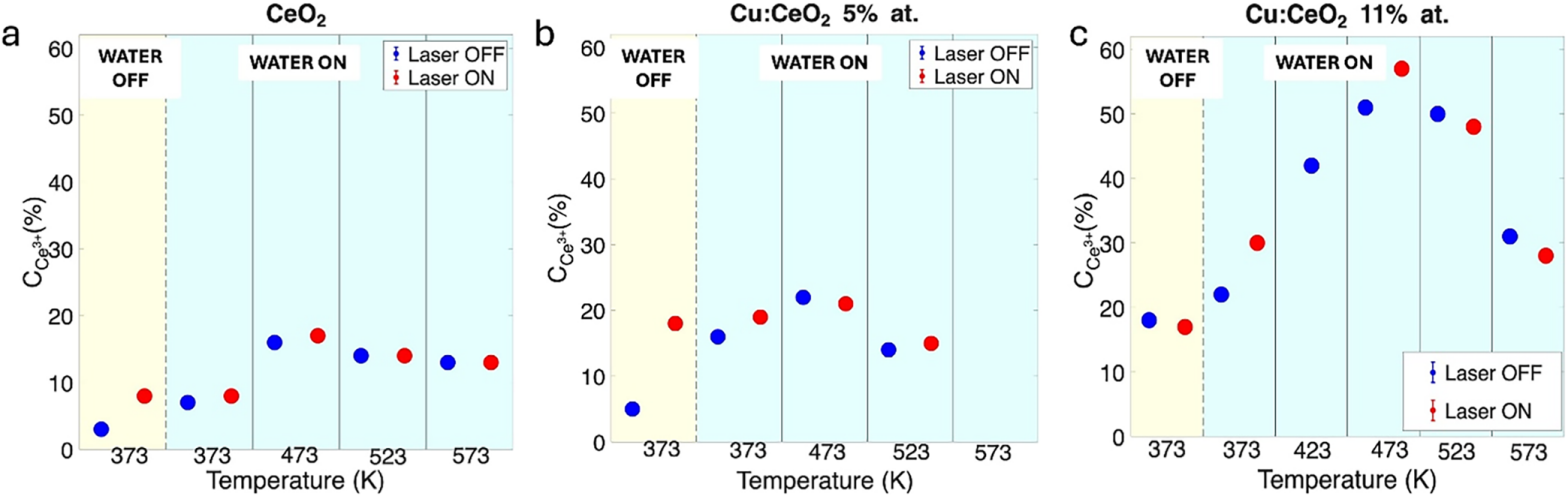
Ce^3+^ concentration of (a) undoped film; (b) Cu:CeO_2_ 5 %; and (c) Cu:CeO_2_ 11 % as a function of temperature and with/without laser illumination. The graphs have been divided into five panels: the one on the left, with a yellow background, reports the Ce^3+^ concentration without water, while the other panels, one for each temperature step, report the concentration of Ce^3+^ ions in water flux. The concentration has been extracted from fitting the Ce M_5_ NEXAFS spectra shown in Figure 3. Error bars (±1% from fit standard deviations) are smaller than marker size.

Figure 3b shows the Ce M_5_ edge NEXAFS spectra for the Cu:CeO_2_ 5 % film in the same conditions as Figure 3a and the Ce^3+^ concentration is reported in Figure 4b. They show an initial reduction in part of the Ce^4+^ ions when the laser is switched on. The effect is similar to the case of the undoped film but more pronounced: in this sample, the laser illumination causes the Ce^3+^ concentration to jump from 5 to 18%. When water is introduced in the reaction chamber—keeping the sample at low temperature—the fraction of reduced ions remains the same within the errors. During the ramp in water, the sample is initially slightly reduced further to 22% and, as observed for the undoped sample, the trend is reverted above 473 K where part of the reduced ions start to be reoxidized. The effect of the laser during the temperature ramp is negligible also in this case. In general, a higher Ce^3+^ concentration is observed in the 5%-doped film as compared to the undoped film, ascribed to a higher reducibility in the presence of Cu.

Figures 3c and 4c show the Ce M_5_ edge NEXAFS spectra and the Ce^3+^ concentration for the Cu:CeO_2_ 11 % film acquired at the different temperatures and laser conditions. The temperature trend followed by the Cu:CeO_2_ 11 % film is similar to the one followed by the other two samples, but more pronounced variations in the Ce^3+^ concentration are observed. Even though the sample starts with a higher Ce^3+^ concentration—due to a slightly different initial condition—after the introduction of light, the fraction of reduced Ce^3+^ ions is the same as for the Cu:CeO_2_ 5 % film, implying that the Ce^3+^ concentration before the ramp is similar in the two doped samples.

The more pronounced reduction of cerium ions with respect to the other two samples is already evident at 373 K when water is introduced in the reaction cell: in the dark, there is a slight increase of the concentration of Ce^3+^ species to 22%, but under light, the concentration increases further to 30%. The trend is continuous up to 473 K: at this temperature, the Ce^3+^ concentration is 51% in the dark and 57% under light illumination. Increasing the temperature from 473 to 523 K, the fraction of reduced ions decreases, while further increasing temperature to 573 K causes a further reoxidation that brings the fraction of Ce^3+^ ions to ∼30%, with an effect of light that is negligible during the reoxidation phase. Even though a partial reduction of the CeO_2_ film due to time-dependent effects, as reported by Shi et al.,^35^ cannot be ruled out, the measurements presented here under comparable acquisition times reveal a clear reduction → oxidation trend across 373–573 K (Figure 4), absent in isothermal conditions. Systematic differences between samples (11% Cu > 5% Cu > undoped) establish photothermal activation and Cu-doping effects beyond time-dependent kinetics alone.

To elucidate the role of Cu dopants in the surface modification process, the NEXAFS spectra at the Cu L_3_ edge were also acquired during the ramp in water flux. Figure 5 shows the Cu L_3_ edge NEXAFS spectra acquired on the two doped samples, normalized by the intensity of the peak at 934 eV ascribed to Cu^1+^ species. For both samples, the NEXAFS spectra have been measured only in few relevant points of the ramp due to the low signal-to-noise ratio. The Cu L_3_ edge NEXAFS spectra of the two samples before starting the ramp (black lines In Figure 5) are very similar. They are both characterized by two main features: a sharp and intense one at ∼930.7 eV and a broader and less intense one at ∼934.2 eV. These components are ascribed to the 2p → 3d transition in Cu^2+^ and Cu^1+^, respectively.^34^ As already observed in ref 3, the two peaks appear shifted by −0.5 and +0.6 eV, respectively, as compared with the reference spectra reported in Figure S2b and in the literature.^36^ These shifts have been previously predicted to occur in similar systems by DFT calculations for XPS^37^ and can be assigned to the different chemical and geometrical environments of Cu atoms incorporated in the CeO_2_ lattice with respect to bulk Cu oxides.^14,38^ Before starting the thermal treatment, the Cu^2+^-related signal is the most intense one in both samples, and the intensity ratio between the two peaks is similar independently of the dopant concentration. In the sample with the lower dopant concentration (Figure 5a), the relative concentration of Cu^2+^ and Cu^1+^ slightly changes during the temperature ramp, from 373 to 523 K, as suggested by the modest decrease in intensity in the shoulder around 931 eV ascribed to the presence of Cu^2+^ ions. On the other hand, the NEXAFS signal of Cu ions in the Cu:CeO_2_ 11 % film (Figure 5b) drastically changes during the temperature ramp, suggesting a complete reduction of the ions. The spectra are consistent with an initial decrease in the Cu^2+^-related feature at 373 K, suggesting possible partial reduction toward Cu^1+^ caused by the combined effect of water in the reaction cell and the laser illumination. During the ramp, a progressively larger fraction of Cu^2+^ ions are reduced to Cu^1+^, reaching a final condition at 523 K in which the signal from Cu^2+^ is almost negligible. Contrary to what happens to the Ce ions, the reduction trend is not reverted at 523 K, and the Cu ions remain in the reduced state until the end of the ramp, at 573 K.

**Figure 5:**
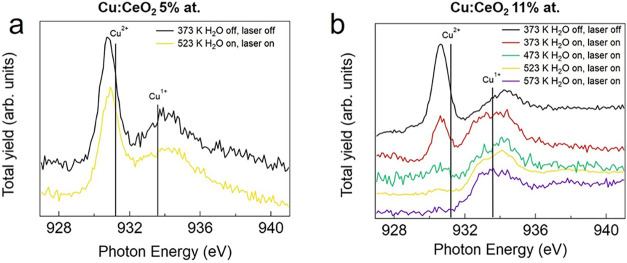
Cu L_3_ edge NEXAFS spectra for (a) Cu:CeO_2_ 5 % and (b) 11 % films acquired at different temperatures in H_2_O flux and normalized at the Cu^1+^ peak. The black lines indicate the bulk reference positions taken from ref 36.

Figure 6 shows the results of the micro-GC analysis of H_2_ on the illuminated samples. Figure 6a,6b, respectively, shows the micro-GC at different temperatures acquired for the Cu:CeO_2_ 5 % and Cu:CeO_2_ 11 % samples. As shown in Figure 6a,6b, the peak ascribed to H_2_ appears at 523 K in the micro-GC, when the Ce starts to be oxidized, increasing in intensity when the temperature is further increased to 573 K when the reduction trend in Ce is reverted (Figures 3b,c and 4b,4c). The micro-GC signal of the undoped CeO_2_ film was also measured during the temperature ramp. Contrary to what was observed with the Cu-doped samples, the undoped film does not show any H_2_-related peak at any temperature, suggesting that pure ceria is not producing detectable amounts of hydrogen when water is introduced in the reaction cell up to 573 K (Figure S3). Figure 6c compares the H_2_-related signal from the doped samples with the signal from the undoped ceria film at 573 K under laser illumination. In the undoped film, the H_2_ production is below the micro-GC detection limit, while the signal is more intense in the sample containing the highest dopant concentration, suggesting that the higher the doping content, the higher the sample reactivity.

**Figure 6:**
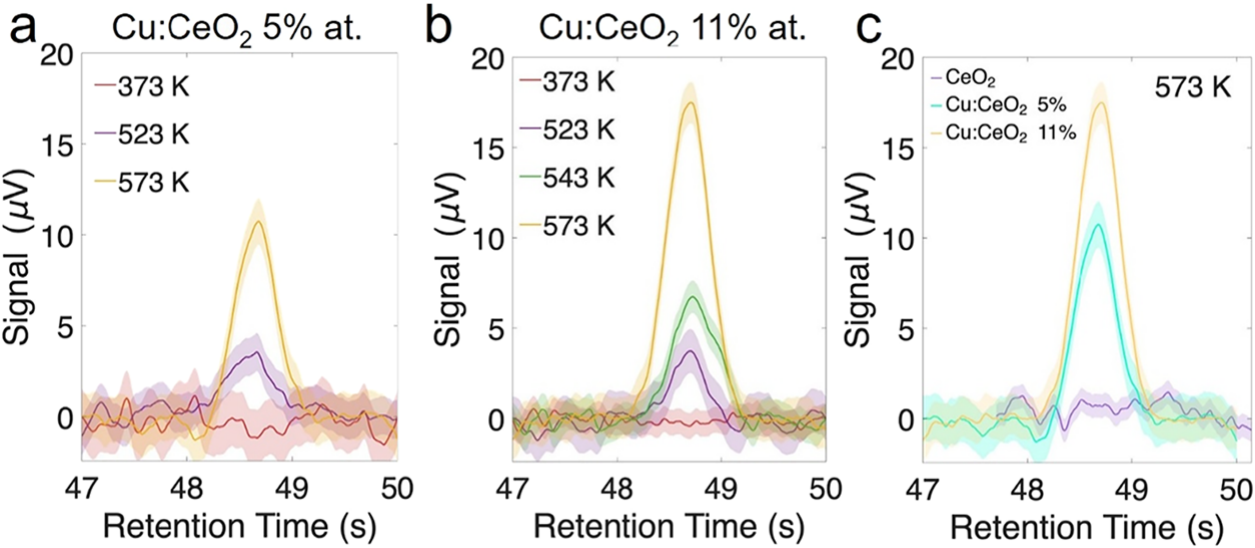
H_2_ micro-GC signal as a function of time for (a) Cu:CeO_2_ 11 % and (b) Cu:CeO_2_ 5 %. (c) Comparison of the H_2_ micro-GC signal for the three different samples, acquired at 573 K under laser illumination and with water fluxed in the reaction cell.

## 4 Discussion

In all the observed samples, the effect of increasing the temperature in water flux and laser illumination is the reduction of part of the Ce ions (Figures 3 and 4) until 473 K, while a further increase in temperature causes the trend to revert and part of the Ce^3+^ ions to be reoxidized back to Ce^4+^. As demonstrated in previous studies on CeO_2_ structures, the presence of Ce^3+^ is commonly a result of the presence of oxygen vacancies,^39–42^ and it is used here as an indirect indicator associated with oxygen vacancy formation, rather than a direct measurement of vacancy concentration. While the reduction trend is similar for all samples, the effect is much more pronounced at higher dopant concentration, where also, the effect of illumination becomes relevant. The doped samples show a similar Cu^1+^/Cu^2+^ ratio at the beginning of the ramp (Figure 5a,5b). During the temperature ramp, with water fluxed in the reaction cell and in the presence of light, the Cu L_3_-edge trends are consistent with Cu^2+^ reduction in both doped samples, partial and mild in the low-doped sample, and almost complete in the 11 % film, for which the Cu^2+^ concentration becomes negligible above 473 K.

Both doped samples show a marked H_2_-related micro-GC peak (Figure 6), which is ascribed to the water splitting reaction taking place on the sample surface. This peak appears under light illumination at 523 K corresponding to the temperature at which Ce^3+^ ions start getting reoxidized to Ce^4+^. On the contrary, the undoped sample shows negligible changes in the micro-GC signal up to 573 K (purple curve in Figure 6c), while the higher the dopant concentration, the higher the intensity of the hydrogen-related peak, suggesting that the presence of Cu is crucial to efficiently split water in this temperature range.

Previous studies on CeO_2_-based systems demonstrated that UV light irradiation can largely increase the material’s catalytic activity and reactive surface area.^43^ This enhancement has been assigned to an increase in Ce^3+^ ion concentration due to the light–matter interaction: UV light irradiation was proven to introduce photoinduced oxygen vacancies (V_O_) in the system.^13,43^ In the samples investigated here, the effect of the interaction with the laser at low temperature is the modification of the film compatibly with the generation of photoinduced V_O_s (Figures 3 and 4). It has been proven that the effects of metallic doping on cerium oxide films are a decrease in the charge recombination probability and a marked decrease in the V_O_ formation energy.^9–11,13,44^ V_O_ forms spontaneously in the vicinity of Cu^2+^,^45^ and a second V_O_ in its neighborhood requires a low formation energy (1.4 eV) reducing surrounding cations to Ce^3+^ and Cu^1+^.^11^ The mechanism proposed in the literature to explain the water splitting process includes a photochemical stage (photoinduced V_O_ formation) and a thermochemical stage (water dissociation).^13,46^ When water is introduced in the reaction cell, H_2_O molecules are chemisorbed at V_O_s. V_O_s participate in the water splitting reaction, by promoting dissociative adsorption of water molecules.^47^ A low energy barrier has to be overcome to dissociate H_2_O, so low that the dissociation of water molecules can be considered extremely easy to occur at low temperature.^13^ In this step, H_2_O dissociates into hydroxyl and hydrogen ion, respectively, located at the position of V_O_ and close to the adjacent oxygen atom.^13^ For exposure at increasing temperature, Ce and Cu ions are progressively reduced, suggesting an increase in the V_O_ concentration up to 473 K. During this process, the effect of light irradiation contributes to the reduction processes commonly associated with oxygen vacancy formation, together with the temperature increase. The presence of water has been observed to favor Ce^4+^ to Ce^3+^ reduction in reduced ceria films, interacting primarily through dissociative adsorption at oxygen vacancy sites forming OH molecules.^13,48–52^ At this point, the two OH bonds have to be broken to form and release H_2_. This step involves a relatively high energy barrier to be overcome and is the limiting step. Above 473 K, temperature is sufficient to break the OH bonding, which leads to Ce^3+^ reoxidation, V_O_ filling, and H_2_ desorption (almost barrier-less).^13,53^ The H_2_ desorption from reduced ceria films for temperatures above 500 K has been previously observed in the literature^54^ and the O_v_ content in the film is expected to increase the H_2_ yield.^54^ While part of the Ce^3+^ ions are reoxidized for temperatures above 473 K, the Cu ions do not reoxidize back to Cu^2+^ and remain in the reduced state. This behavior is coherent with the fact that the Ce^4+^/Ce^3+^ couple is more favorable for oxidation than the Cu^2+^/Cu^1+^ couple, which has a lower standard electron potential.^55,56^

The effective role of copper doping is evident in the differences in the H_2_ production observed in the micro-GC for the three different samples (Figure 6c). In the pure ceria film, the H_2_ production is negligible because of the low concentration of V_O_s generated by the light and temperature (Figure 4a). The mild reoxidation of part of the Ce^3+^ ions observed in the NEXAFS signal suggests that bare CeO_2_ is not inert but that the H_2_ production is too low to be detected with the micro-GC. The presence of Cu dopants is expected to modify the crystal structure of CeO_2_, reducing the oxygen vacancy formation energy.^16^ The higher the dopant concentration, the higher the concentration of V_O_ sites, where water molecules adsorb and dissociate,^45^ and the higher the amount of H_2_ produced. On the other side, Cu does not modify the temperature that activates hydrogen production (at around 523 K) when Ce starts being reoxidized. The correlation between the increase of the Ce^3+^ fraction, the spectral changes at the Cu L_3_-edge, and the enhanced H_2_ signal suggests a cooperative redox mechanism, in which both cerium and copper sites contribute to the processes leading to hydrogen formation.

## 5 Conclusions

Pure and Cu-doped CeO_2_ films of 5 nm thickness were investigated to elucidate the evolution of Ce and Cu oxidation states during photothermal activation under 1 bar of water flux, using laser irradiation at 375 nm, at the onset of the optical absorption edge of the films. By integrating advanced characterization techniques such as ambient-pressure NEXAFS and micro-GC, we provide direct evidence of the decisive role of Cu in the process of water dissociation and H_2_ production through the promotion of oxygen vacancy formation. For all the investigated samples, the temperature increase in water flux and light illumination initially promotes the reduction of Ce^4+^ to Ce^3+^, assigned to an increase of V_O_ concentration, while above 473 K, the trend is reversed and part of the reduced ions are reoxidized to Ce^4+^. This inversion is observed in undoped and doped films, occurring at the same temperature, but becomes more pronounced as the Cu concentration increases. In the highly doped sample (11 %), the Ce^3+^ fraction reaches values above 50% before being reoxidized at higher temperature, coinciding with the onset of detectable H_2_ production. At the same time, in both doped samples, Cu ions are reduced; however, this reduction is very mild in the low-doped sample, while in the 11 % Cu-doped film, a progressive and almost complete reduction of Cu^2+^ takes place during the temperature ramp. In both cases, the Cu^2+^ reduction is not reverted above 473 K. Micro-GC analysis confirms that hydrogen formation is negligible on pure ceria under the explored conditions, whereas Cu doping increases the reactivity of the film in water, as proven by the increase in the hydrogen-related signal at higher dopant concentration, suggesting an increase in the oxygen vacancy concentration in the material. The correlation between the hydrogen production and the Cu concentration, together with the inversion in the Ce^3+^/Ce^4+^ ratio in the doped samples, suggests the fundamental role of dopants in facilitating oxygen vacancy formation in the oxide. Unlike thermal H_2_ treatments,^3^ photothermal H_2_O activation reveals dynamic Ce^3+^/Ce^4+^ cycling correlated with H_2_ production, establishing Cu-doping synergy under catalytic conditions. The findings of this study provide a mechanistic understanding of the role of Cu doping in modulating the electronic properties of CeO_2_ thin films and, as a consequence, the catalytic activity. Further exploration of light-driven dynamics of charge transfers at the atomic level could pave the way for improved, more efficient, and cost-effective materials for hydrogen production.

## Supplementary Material



## Notes

Details of the XPS spectra and fitting of the three samples, references for the XANES Cu L_3_ and Ce M_5_ edges, and micro-GC signal from the bare CeO_2_ film (PDF)

## References

[1] Zhang Z., Liu J., Gu J., Su L., Cheng L. (2014). An Overview of Metal
Oxide Materials as Electrocatalysts and Supports for Polymer Electrolyte
Fuel Cells. Energy Environ. Sci..

[2] Catalysis by Ceria and Related Materials | Catalytic Science Series. https://www.worldscientific.com/worldscibooks/10.1142/p249.

[3] Vikatakavi A., Mauri S., Rivera-Salazar M. L., Dobovičnik E., Pelatti S., D’Addato S., Torelli P., Luches P., Benedetti S. (2024). Role of Metal
Dopants in Hydrogen Dissociation on Cu:CeO2
and Fe:CeO2 Surfaces Studied by Ambient-Pressure X-Ray Absorption
Spectroscopy. ACS Appl. Energy Mater..

[4] Kazazi M., Moradi B., Delshad Chermahini M. (2019). Enhanced Photocatalytic Degradation
of Methyl Orange Using Ag/Sn-Doped CeO_2_ Nanocomposite. J. Mater. Sci.: Mater. Electron..

[5] Xu Y., Mofarah S. S., Mehmood R., Cazorla C., Koshy P., Sorrell C. C. (2021). Design Strategies
for Ceria Nanomaterials: Untangling
Key Mechanistic Concepts. Mater. Horiz..

[6] Patsalas P., Logothetidis S., Sygellou L., Kennou S. (2003). Structure-Dependent
Electronic Properties of Nanocrystalline Cerium Oxide Films. Phys. Rev. B.

[7] Zhang X., Zhu L., Hou Q., Guan J., Lu Y., Keal T. W., Buckeridge J., Catlow C. R. A., Sokol A. A. (2023). Toward
a Consistent
Prediction of Defect Chemistry in CeO_2_. Chem. Mater..

[8] Pelli
Cresi J. S., Di Mario L., Catone D., Martelli F., Paladini A., Turchini S., D’Addato S., Luches P., O’Keeffe P. (2020). Ultrafast Formation of Small Polarons
and the Optical Gap in CeO_2_. J. Phys.
Chem. Lett..

[9] Righi G., Benedetti S., Magri R. (2022). Investigation of the
Structural and
Electronic Differences between Silver and Copper Doped Ceria Using
the Density Functional Theory. J. Phys.: Condens.
Matter.

[10] Benedetti S., Righi G., Luches P., D’Addato S., Magri R., Selloni A. (2020). Surface Reactivity of Ag-Modified
Ceria to Hydrogen: A Combined Experimental and Theoretical Investigation. ACS Appl. Mater. Interfaces.

[11] Vikatakavi A., Benedetti S., Righi G., Magri R., D’Addato S., Luches P., Selloni A. (2022). Interaction of Hydrogen with Cu-Modified
Cerium Oxide Surfaces. J. Phys. Chem. C.

[12] Yang Z., He B., Lu Z., Hermansson K. (2010). Physisorbed, Chemisorbed, and Oxidized
CO on Highly Active Cu–CeO_2_(111). J. Phys. Chem. C.

[13] Li R., Wen C., Yan K., Liu T., Zhang B., Xu M., Zhou Z. (2023). The Water Splitting
Cycle for Hydrogen Production at Photo-Induced
Oxygen Vacancies Using Solar Energy: Experiments and DFT Calculation
on Pure and Metal-Doped CeO_2_. J.
Mater. Chem. A.

[14] Lu Z., Yang Z., He B., Castleton C., Hermansson K. (2011). Cu-Doped Ceria: Oxygen Vacancy Formation
Made Easy. Chem. Phys. Lett..

[15] Yan K., Wen C., Li R., Zhang B., Liu T., Liu Q., Zhou Z. (2023). Morphological
Optimized CeO_2_ and Cu-Doped CeO_2_ Nanocrystals
for Hydrogen Production by Solar Photo-Thermochemical
Water Splitting Based on Surface Photoinduced Oxygen Vacancies. Appl. Surf. Sci..

[16] Gasperi G., Brugnoli L., Pedone A., Menziani M. C., Valeri S., Luches P. (2019). Reducibility of Ag- and Cu-Modified
Ultrathin Epitaxial
Cerium Oxide Films. J. Phys. Chem. C.

[17] Monte M., Munuera G., Costa D., Conesa J. C., Martínez-Arias A. (2015). Near-Ambient
XPS Characterization of Interfacial Copper Species in Ceria-Supported
Copper Catalysts. Phys. Chem. Chem. Phys..

[18] Konsolakis M. (2016). The Role of
Copper–Ceria Interactions in Catalysis Science: Recent Theoretical
and Experimental Advances. Appl. Catal., B.

[19] Hermes E. D., Jenness G. R., Schmidt J. R. (2015). Decoupling
the Electronic, Geometric
and Interfacial Contributions to Support Effects in Heterogeneous
Catalysis. Mol. Simul..

[20] Beckers J., Rothenberg G. (2008). Redox Properties
of Doped and Supported Copper–Ceria
Catalysts. Dalton Trans..

[21] Tang X., Zhang B., Li Y., Xu Y., Xin Q., Shen W. (2005). CuO/CeO_2_ Catalysts: Redox
Features and Catalytic Behaviors. Appl. Catal.,
A.

[22] Papadopoulos C., Kappis K., Papavasiliou J., Vakros J., Kuśmierz M., Gac W., Georgiou Y., Deligiannakis Y., Avgouropoulos G. (2020). Copper-Promoted
Ceria Catalysts for CO Oxidation Reaction. Catal.
Today.

[23] Righi G., Magri R., Selloni A. (2019). H2 Dissociation on Noble Metal Single
Atom Catalysts Adsorbed on and Doped into CeO_2_ (111). J. Phys. Chem. C.

[24] Chen A., Yu X., Zhou Y., Miao S., Li Y., Kuld S., Sehested J., Liu J., Aoki T., Hong S., Camellone M. F., Fabris S., Ning J., Jin C., Yang C., Nefedov A., Wöll C., Wang Y., Shen W. (2019). Structure
of the Catalytically Active
Copper–Ceria Interfacial Perimeter. Nat.
Catal.

[25] Silversmit G., Poelman H., Balcaen V., Heynderickx P. M., Olea M., Nikitenko S., Bras W., Smet P. F., Poelman D., De Gryse R., Reniers M.-F., Marin G. B. (2009). In-Situ
XAS Study on the Cu and Ce Local Structural Changes in a CuO–CeO_2_/Al_2_O_3_ Catalyst under Propane Reduction
and Re-Oxidation. J. Phys. Chem. Solids.

[26] Oguchi H., Nishiguchi T., Matsumoto T., Kanai H., Utani K., Matsumura Y., Imamura S. (2005). Steam Reforming of Methanol over
Cu/CeO_2_/ZrO_2_ Catalysts. Appl. Catal., A.

[27] Castán-Guerrero C., Krizmancic D., Bonanni V., Edla R., Deluisa A., Salvador F., Rossi G., Panaccione G., Torelli P. (2018). A Reaction Cell for
Ambient Pressure Soft X-Ray Absorption
Spectroscopy. Rev. Sci. Instrum..

[28] Luches P., Pagliuca F., Valeri S. (2011). Morphology,
Stoichiometry, and Interface
Structure of CeO_2_ Ultrathin Films on Pt(111). J. Phys. Chem. C.

[29] Grioni M., van Acker J. F., Czyžyk M. T. (1992). Unoccupied Electronic
Structure and Core-Hole Effects in the x-Ray-Absorption Spectra of
Cu_2_O. Phys. Rev. B.

[30] Stöhr, J. NEXAFS Spectroscopy. In Springer Series in Surface Sciences; Ertl, G. ; Gomer, R. ; Mills, D. L. ; Lotsch, H. K. V. , Eds.; Springer: Berlin, Heidelberg, 1992; Vol. 25.

[31] Spurio E., Pelatti S., D’Addato S., Luches P. (2024). Plasmonic Properties
and Stability of Au and Cu Nanoparticles Embedded in Cerium Oxide. J. Phys.: Condens. Matter.

[32] Cresi J. S. P., Spadaro M. C., D’Addato S., Valeri S., Benedetti S., Bona A. D., Catone D., Mario L. D., O’Keeffe P., Paladini A., Bertoni G., Luches P. (2019). Highly Efficient Plasmon-Mediated
Electron Injection into Cerium Oxide from Embedded Silver Nanoparticles. Nanoscale.

[33] Idriss H. (2026). Extraction
of Band Gap Energies and Composition of Mixed-Phase Polycrystalline
Semiconductors; a Possible Alternative Method. J. Phys.: Condens. Matter.

[34] Kucheyev S. O., Clapsaddle B. J., Wang Y. M., van Buuren T., Hamza A. V. (2007). Electronic Structure
of Nanoporous Ceria from X-Ray
Absorption Spectroscopy and Atomic Multiplet Calculations. Phys. Rev. B.

[35] Shi Y., Wang L., Wang Z., Vinai G., Braglia L., Torelli P., Aruta C., Traversa E., Liu W., Yang N. (2021). Defect Engineering for Tuning the Photoresponse of Ceria-Based Solid
Oxide Photoelectrochemical Cells. ACS Appl.
Mater. Interfaces.

[36] Cheang-Wong J. C., Ortega C., Siejka J., Ortiz C., Sacchi M., Carniato S., Dufour G., Rochet F., Roulet H. (1993). Study of CuOy
Layers on Si and MgO by a Combination of Ion Beam Analysis (RBS/NRA),
X-Ray Photoemission Spectroscopy (XPS) and X-Ray Absorption Spectroscopy
(XAS). Appl. Surf. Sci..

[37] Benedetti S., Nilius N., Valeri S., Tosoni S., Albanese E., Pacchioni G. (2016). Dopant-Induced
Diffusion Processes at Metal–Oxide
Interfaces Studied for Iron- and Chromium-Doped MgO/Mo(001) Model
Systems. J. Phys. Chem. C.

[38] Braglia L., Tavani F., Mauri S., Edla R., Krizmancic D., Tofoni A., Colombo V., D’Angelo P., Torelli P. (2021). Catching the Reversible Formation
and Reactivity of
Surface Defective Sites in Metal–Organic Frameworks: An Operando
Ambient Pressure-NEXAFS Investigation. J. Phys.
Chem. Lett..

[39] Esch F., Fabris S., Zhou L., Montini T., Africh C., Fornasiero P., Comelli G., Rosei R. (2005). Electron Localization
Determines Defect Formation on Ceria Substrates. Science.

[40] Zhang F., Chan S.-W., Spanier J. E., Apak E., Jin Q., Robinson R. D., Herman I. P. (2002). Cerium Oxide Nanoparticles: Size-Selective
Formation and Structure Analysis. Appl. Phys.
Lett..

[41] Deshpande S., Patil S., Kuchibhatla S. V., Seal S. (2005). Size Dependency Variation
in Lattice Parameter and Valency States in Nanocrystalline Cerium
Oxide. Appl. Phys. Lett..

[42] Dutta P., Pal S., Seehra M. S., Shi Y., Eyring E. M., Ernst R. D. (2006). Concentration
of Ce^3+^ and Oxygen Vacancies in Cerium Oxide Nanoparticles. Chem. Mater..

[43] Wu T.-S., Syu L.-Y., Lin C.-N., Lin B.-H., Liao Y.-H., Weng S.-C., Huang Y.-J., Jeng H.-T., Lu S.-Y., Chang S.-L., Soo Y.-L. (2019). Enhancement
of Catalytic Activity
by UV-Light Irradiation in CeO2 Nanocrystals. Sci. Rep..

[44] Sartoretti E., Novara C., Paganini M. C., Chiesa M., Castellino M., Giorgis F., Piumetti M., Bensaid S., Fino D., Russo N. (2023). Investigation of Cu-Doped Ceria through
a Combined Spectroscopic
Approach: Involvement of Different Catalytic Sites in CO Oxidation. Catal. Today.

[45] Wang X., Rodriguez J. A., Hanson J. C., Gamarra D., Martínez-Arias A., Fernández-García M. (2006). In Situ Studies
of the Active Sites
for the Water Gas Shift Reaction over Cu–CeO_2_ Catalysts:
Complex Interaction between Metallic Copper and Oxygen Vacancies of
Ceria. J. Phys. Chem. B.

[46] Henderson M. A., Perkins C. L., Engelhard M. H., Thevuthasan S., Peden C. H. F. (2003). Redox Properties of Water on the
Oxidized and Reduced
Surfaces of CeO_2_(111). Surf. Sci..

[47] Menetrey M., Markovits A., Minot C. (2003). Reactivity of a Reduced Metal Oxide
Surface: Hydrogen, Water and Carbon Monoxide Adsorption on Oxygen
Defective Rutile TiO_2_(110). Surf.
Sci..

[48] Fronzi M., Piccinin S., Delley B., Traversa E., Stampfl C. (2009). Water Adsorption
on the Stoichiometric and Reduced CeO_2_(111) Surface: A
First-Principles Investigation. Phys. Chem.
Chem. Phys..

[49] Zhou Q., Akber H., Zhao A., Yang F., Liu Z. (2023). Interaction
of Water with Ceria Thin Film. ChemCatChem.

[50] Matolín V., Matolínová I., Dvořák F., Johánek V., Mysliveček J., Prince K. C., Skála T., Stetsovych O., Tsud N., Václavů M., Šmíd B. (2012). Water Interaction with CeO_2_(111)/Cu­(111)
Model Catalyst Surface. Catal. Today.

[51] Molinari M., Parker S. C., Sayle D. C., Islam M. S. (2012). Water Adsorption
and Its Effect on the Stability of Low Index Stoichiometric and Reduced
Surfaces of Ceria. J. Phys. Chem. C.

[52] Berner U., Schierbaum K., Jones G., Wincott P., Haq S., Thornton G. (2000). Ultrathin
Ordered CeO_2_ Overlayers on Pt(111):
Interaction with NO_2_, NO, H_2_O and CO. Surf. Sci..

[53] Wu Q., Li Z., Zhang X., Huang W., Ni M., Cen K., Zhang Y. (2021). Enhanced Defect-Water Hydrogen Evolution Method for
Efficient Solar
Utilization: Photo-Thermal Chemical Coupling on Oxygen Vacancy. Chem. Eng. J..

[54] Chen B., Ma Y., Ding L., Xu L., Wu Z., Yuan Q., Huang W. (2013). Reactivity of Hydroxyls and Water
on a CeO_2_(111) Thin
Film Surface: The Role of Oxygen Vacancy. J.
Phys. Chem. C.

[55] Rao G. P., Murthy A. R. V. (1964). Measurement of
Formal Oxidation—Reduction Potentials
of Cerium­(IV)—Cerium­(III) System in Acetonitrile. J. Phys. Chem. A.

[56] Bard, A. J. ; Parsons, R. ; Jordan, J. Standard Potentials in Aqueous Solution; Routledge, 2017.

