# Supplementary material for: Ambient-Pressure Near-Edge X‑ray Absorption Fine Structure Study of the Photothermal Water Splitting Process on the Cu:CeO2 Nanostructure Surface
Source: ACS Appl Nano Mater. 2026 May 13;9(20):9400–8. doi: 10.1021/acsanm.6c00868 (PMC13206250; doi:10.1021/acsanm.6c00868)
Supplement: Supplementary file 1 [file an6c00868_si_001.pdf]

Supporting Information

Ambient Pressure Near-Edge X-Ray Absorption Fine Structure Study of Photothermal Water Splitting Process on Cu:CeO_2 Nanostructure Surface

Eleonora Spurio[1], Silvia Mauri[2,3*], Samuele Pelatti[1], Mario Leopoldo Rivera-Salazar[2,4] Sergio D'Addato[1,5], Piero Torelli[2], Paola Luches[1], and Stefania Benedetti[1]*

[1]Istituto Nanoscienze – CNR, S3, Modena 41125, Italy

[2]CNR-IOM, Laboratorio TASC, Basovizza, Trieste 34149, Italy

[3] MAX IV Laboratory, Lund University, SE-221 00 Lund, Sweden

[4] Dipartimento di Fisica, Università di Trieste, Trieste 34127, Italy

[5] Dipartimento di Scienze Fisiche, Informatiche, Matematiche, Università di Modena e Reggio Emilia, Modena 41125, Italy

** these authors contributed equally*

Corresponding author: eleonora.spurio@cnr.it

1. X-ray photoemission spectra

Pure ceria and Cu doped CeO_2 were characterized right after the deposition on MgO by in situ XPS using Al Kα photons. In this work, the Ce^{3+} concentration has been estimated by fitting the Ce 3d XPS lines with Ce^{3+}- and Ce^{4+}-related components, following the procedure introduced by Skala et al[1]. From the results of the fitting procedure – shown together with the spectra in Figure S3 – 100% of Ce ions are in the Ce^{4+} state for all films.

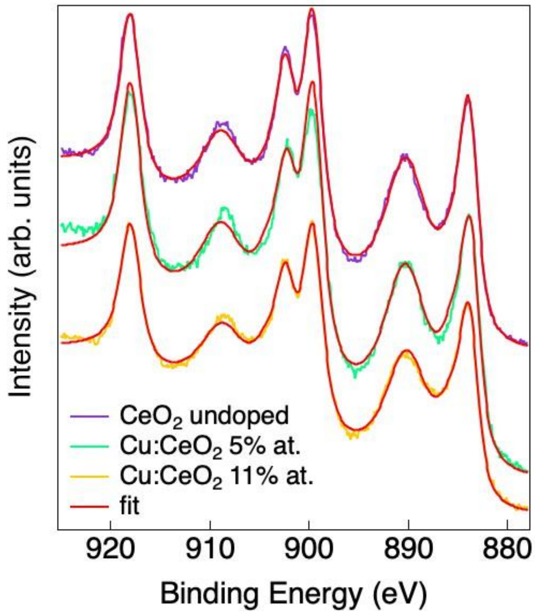

Figure S1 Ce 3d XPS spectra of the measured samples. The red curves in b) are the results of the fitting procedure

2. Reference NEXAFS spectra

The reference Ce $M_{4,5}$ spectra on CeO_2 and Ce_2O_3 were obtained on commercial powders from Umicore and measured after controlled oxidation and reduction cycles in the reaction cell. The Cu L_3 reference spectra were measured in the same reaction cell at the APE-HE beamline from commercial CuO, Cu_2O, and Cu powders purchased by Sigma-Aldrich.

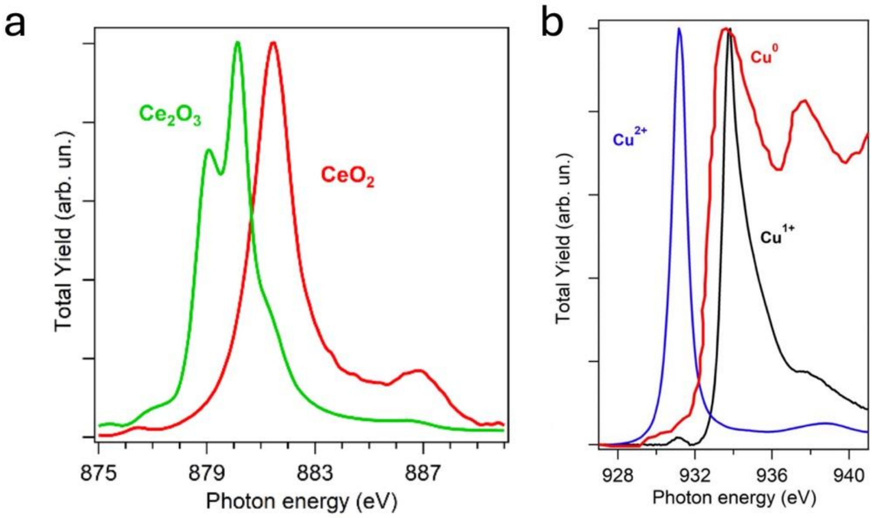

Figure S2 Reference spectra measured for a) CeO_2 and Ce_2O_3 and b) Cu, CuO, and Cu_2O

3. micro-GC signal from pure CeO_2

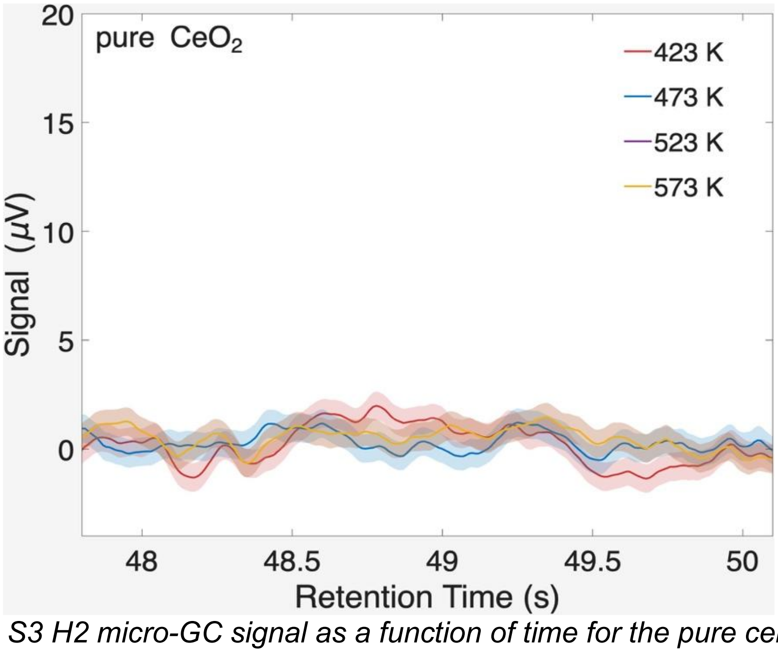

Figure S3 H2 micro-GC signal as a function of time for the pure ceria film

(1) Skála, T.; Šutara, F.; Škoda, M.; Prince, K. C.; Matolín, V. Palladium Interaction with CeO2, Sn–Ce–O and Ga–Ce–O Layers. *J. Phys.: Condens. Matter* **2008**, *21* (5), 055005.
